# Impact of COVID-19 Crisis in the Management of Diabetic Foot Patients in King Abdulaziz University Hospital, Jeddah, Saudi Arabia

**DOI:** 10.7759/cureus.36613

**Published:** 2023-03-24

**Authors:** Rawan T Harun, Abdullah A Almohammadi, Maryam M Alnashri, Sarah Alsamiri, Maram Alkhatieb

**Affiliations:** 1 General Surgery, Faculty of Medicine, King Abdulaziz University, Jeddah, SAU; 2 Surgery, Faculty of Medicine, King Abdulaziz University, Jeddah, SAU; 3 Internal Medicine, Faculty of Medicine, King Abdulaziz University, Jeddah, SAU

**Keywords:** surgery general, diabetic foot complications, diabetic foot management, lower limb amputation, major limb amputation, covid-19 outbreak

## Abstract

Background

The COVID-19 pandemic has a heavy burden on the approach of diabetic foot care worldwide. We aim to determine the impact of the COVID-19 outbreak on patients with diabetic foot (DF).

Materials and methods

This population-based cohort study included all patients diagnosed with the diabetic foot from 2019-2020 (pre-lockdown) and 2020-2021 (post-lockdown) in a tertiary center of Jeddah, Saudi Arabia.

Results

Among all the participants (n=358), a non-significant difference was found between amputation rate during and before the COVID-19 pandemic (P-value=0.0983). Also, it showed a significantly higher percentage of patients who had acute lower limb ischemia compared to those having it before the pandemic (P-value=0.029).

Conclusions and relevance

In conclusion, our study found that the COVID-19 pandemic was not associated with excess amputations along with mortality rate, as the management during the pandemic showed adequate diabetic foot care by improving the prevention methods through hospital protocol restrictions and facilitating access to virtual clinics.

## Introduction

Diabetic foot ulcers (DFUs) are the leading cause of non-traumatic lower extremity amputation worldwide [1], and it is considered the most common indication for admission in diabetic patients [2]. DFUs represent the most severe complications in diabetes mellitus.

DFUs significantly impact the patient's morbidity, mortality, and socioeconomic status. In addition, it has a heavy burden on the health care system, which requires a long time of care and hospitalization. Despite the seriousness of this disease, proper and early management with a multidisciplinary approach can prevent the most severe consequence by reducing the amputation rate by more than 50% [3].

During the peak of the coronavirus disease 2019 (COVID-19) pandemic, patients with diabetes mellitus and diabetic foot ulcers were facing a considerable challenge. This pandemic has significantly affected most countries' health care system and economy. As a result, many countries decided to lock down to prevent the further spread of the virus. This lockdown directly and significantly impacted those patients with diabetes mellitus or its complications. Many clinics had to cancel their appointments interfering with regular follow-ups with DFU patients. Interruption of early diagnosis or interventions may have led to increased hospitalization of patients with severe DFU at high amputation risk [4].

The impact of COVID-19 was global research attention and studies from different aspects since it is highly contagious and can cause enormous social disharmony and economic loss. However, though the relationship of COVID-19 with diabetes is under extensive evaluation, its specific impact on the diabetic foot remains sparsely studied.

We aimed to determine the impact of the COVID-19 outbreak on patients with DFUs by conducting a study on diabetic foot complications and mortality during the pandemic in comparison to the pre-pandemic period.

## Materials and methods

This is a retrospective study held at King Abdulaziz University Hospital (KAUH) to review data from 2019-2020 (pre-lockdown) and 2020-2021 (post-lockdown). KAUH is a tertiary training institution and perhaps one of the best medical centers in the western region of Saudi Arabia.

All patients who had previously been diagnosed with diabetes (DM) and were diagnosed with diabetic foot ulcers or foot gangrene (dry/wet) were included in this study. The surgical team made the diagnosis based on the clinical presentation and test results. Due to the study's retrospective nature, informed consent is not required. The institutional review board at KAUH granted ethical approval (Reference No. 542-20).

A total of 358 patients' records were included using Excel (Microsoft® Corp., Redmond, WA). Descriptive data are age, date of birth, and gender. Admission date, the number of admissions regarding diabetes, duration of diabetes, site of admission, comorbidities, foot infection, previous surgeries, history of peripheral artery disease (PAD) revascularization, amputation history, ICU admission outcome, and cause of death were recorded. Then we divided amputations according to their date and whether it was major or minor amputations; below-knee amputation and above-knee amputation were considered major, while toe amputations and trans-metatarsal amputations were considered minor.

We determined the impact of COVID-19 during the lockdown period in Jeddah from March 2020 to September 2020 by comparing the above variables to the same period a year before, from March 2019 to September 2019.

The correlation test was carried out using SPSS version 24 (IBM Corp., Armonk, NY). A p-value of 0.05 is considered significant. Subsequently, a logistic regression was performed on the variable data set to evaluate delay and attribution factors.

Data analysis

Data were analyzed statistically using SPSS version 26 (IBM Corp., Armonk, NY). Qualitative data were expressed as numbers and percentages, and the Chi-squared test (χ2) was applied to test the relationship between variables. Quantitative data were expressed as mean and standard deviation (Mean ± SD), where the Mann-Whitney test was used for non-parametric variables. A p-value of less than 0.05 was considered statistically significant.

## Results

The mean age of the participants was 63.19 ± 14.72 years during the COVID-19 pandemic and 64.5 ± 13.4 years before the pandemic. Seventy-four (53.6%) and 64 (46.4%) of the participants had Saudi nationality during and before the COVID-19 pandemic, respectively. Further, patients with lower creatinine level had a significantly higher percent during the COVID-19 pandemic (p=< 0.05). While a non-significant difference was found between other patients’ characters, clinical data, lab results, or chronic diseases during and before the COVID-19 pandemic (p=> 0.05) (Table 1).

**Table 1:** Difference between patients’ characters, clinical data, lab results, and chronic diseases during and before the COVID-19 pandemic DM: Diabetes mellitus; CRP: C-reactive protein; CVD: Cardiovascular disease; HTN: Hypertension; CKD: Chronic kidney disease.

Variable	During COVID-19 pandemic (No.: 152)	Before COVID-19 pandemic (No.: 206)	p-value
	No. (%)	No. (%)	
Age	63.19 ± 14.72	64.5 ± 13.4	0.384
Gender			
Female	57 (47.5)	63 (52.5)	
Male	95 (39.9)	143 (60.1)	0.171
Nationality			
Non-Saudi	78 (35.5)	142 (64.5)	
Saudi	74 (53.6)	64 (46.4)	0.001
Admission number due to DM	2.07 ± 1.47	2.29 ± 1.79	0.289
Duration of Diabetes	18 ± 8.47	15.97 ± 8.44	0.211
Ulcer number	1.02 ± 0.8	0.86 ± 0.74	0.073
Ulcer duration/days	61.17 ± 110.5	63.54 ± 83.95	0.619
Number of surgeries	1.34 ± 1.24	1.12 ± 1.21	0.073
BMI	27.74 ± 7.16	27.51 ± 6.35	0.945
WBC	16.24 ± 11.31	17.37 ± 9.97	0.284
Hg	9.91 ± 2.47	9.73 ± 2.5	0.425
HbA1C	9.12 ± 2.63	9.46 ± 5.69	0.863
CRP	146.16 ± 96.25	141.34 ± 82.71	0.868
D-dimer	6.57 ± 10.93	4.02 ± 5.55	0.852
Creatinine	157.52 ± 165.22	199.4 ± 206.39	0.004
Type of diabetes:			
Type 1	26 (40)	39 (60)	
Type 2	114 (43.5)	148 (56.5)	
Unknown	12 (38.7)	19 (61.3)	0.795
Site of admission:			
ER	130 (43.9)	166 (56.1)	0.471
N/A	2 (33.3)	4 (66.7)	
Outpatient	20 (35.7)	36 (64.3)	
Treatment:			
Oral Hypoglycemic Agents	41 (45.6)	49 (54.4)	0.482
Insulin treatment	44 (42.3)	60 (57.7)	0.853
Chronic diseases:			
CVD	47 (38.5)	75 (61.5)	0.279
HTN	114 (41.9)	158 (58.1)	0.71
CKD	26 (38.2)	42 (61.8)	0.434
Diabetic nephropathy	4 (28.6)	10 (71.4)	0.284
Diabetic neuropathy	7 (70)	3 (30)	0.074
Malignancy	2 (40)	3 (60)	0.911
More than one	50 (38.5)	80 (61.5)	0.248
Chronic disease	24 (53.3)	21 (46.7)	0.114

Table 2 shows that during the COVID-19 pandemic, there was a significantly higher percentage of patients who had acute lower limb (LL) ischemia compared to those having it before the pandemic (p=> 0.05). On the other hand, a non-significant difference was found between gangrene, ulcer characters, foot infection, necrotizing fasciitis, osteomyelitis and surgical history during and before the COVID-19 pandemic (p=> 0.05).

**Table 2:** Difference between patients’ gangrene, ulcer characters, ischemia, foot infection, necrotizing fasciitis, osteomyelitis and surgical history during and before the COVID-19 pandemic LL: Lower limb; PAD: Peripheral artery disease.

Variable	During COVID-19 pandemic (No.: 152)	Before COVID-19 pandemic (No.: 206)	p-value
	No. (%)	No. (%)	
Gangrene:			
Dry	106 (42.9)	141 (57.1)	0.794
Wet	118 (44)	159 (56)	0.299
Gas	139 (42.5)	188 (57.5)	0.951
Chronic Ulcer:			
No	69 (42.1)	11 (57.2)	0.892
Yes	83 (42.8)	95 (57.9)	
Ulcer type :			
N/A	138 (41.7)	193 (58.3)	0.835
Ischemic	8 (57.1)	6 (42.9)	
Neuro-ischemic	2 (50)	2 (50)	
Neuropathic	1 (50)	1 (50)	
Pressure Ulcer	3 (42.9)	4 (57.1)	
Acute LL ischemia			0.029
No	141 (41.2)	201 (58.8)	
Yes	11 (68.8)	5 (31.3)	
Necrotizing Fasciitis			0.532
No	144 (42.1)	198 (57.9)	
Yes	8 (50)	8 (50)	
Foot infection			0.407
No	35 (46.7)	40 (53.3)	
Yes	117 (41.3)	166 (58.7)	
Osteomyelitis			0.125
No	126 (44.5)	157 (55.5)	
Yes	26 (34.7)	49 (65.3)	
Surgical history			
No	69 (40.6)	101 (59.4)	0.469
Yes	83 (44.1)	105 (55.9)	
Number of PAD revascularization	1.5 ± 0.67	1.42 ± 0.68	0.509

Table 3 demonstrated that a total of 129 (42.4%) and 175 (57.6%) of the participants in both groups underwent amputation. Among all the participants, 50 (41%) underwent major amputations during the pandemic compared to those before the COVID-19 pandemic which calculated as 72 (59%). Moreover, the most common cause of amputation was infection, 42.2% and 57.8% during and before the COVID-19 pandemic, respectively, and peripheral vascular disease was present in 63 (38.2%) and 102 (61.8%) of the participants in both groups. Further, a non-significant difference was found between amputation rate during and before the COVID-19 pandemic (p=> 0.05).

**Table 3:** Difference between patients’ amputation data during and before the COVID-19 pandemic. ESRD: End-stage renal disease

Variable	During COVID-19 pandemic (No.: 152)	Before COVID-19 pandemic (No.: 206)	p-value
	No. (%)	No. (%)	
Amputation			
No	23 (42.6)	31 (57.4)	0.0983
Yes	129 (42.4)	175 (57.6)	
Amputation			
Minor	50 (43.1)	66 (56.9)	0.933
Major	50 (41)	72 (59)	
Both	30 (45.5)	36 (54.5)	
No	22 (40.7)	32 (59.3)	
Number of amputations	1.38 ± 0.94	1.31 ± 0.83	0.689
Number of minor amputations	0.72 ± 0.81	0.64 ± 0.76	0.453
Level of minor amputation:			
Great toes or first ray	17 (34.7)	32 (65.3)	0.694
N/A	85 (43.6)	110 (56.4)	
Other toes	26 (47.3)	29 (52.7)	
Through ankle or Taurus	1 (25)	3 (75)	
Through metatarsal bones	20 (44.4)	25 (55.6)	
Through tarsometatarsal joints	3 (30)	7 (70)	
Number of major amputations	0.67 ± 0.75	0.7 ± 0.75	0.73
Level of major amputation			
Above knee	57 (46.7)	65 (53.3)	0.279
Below Knee	26 (34.7)	49 (65.3)	
Hip	0 (0.0)	2 (100)	
Through knee	0 (0.0)	1 (100)	
N/A	69 (43.7)	89 (56.3)	
Cause of amputations			
Critical ischemia	11 (45.8)	13 (54.2)	0.943
Infection	76 (42.2)	104 (57.8)	
Trauma	5 (35.7)	9 (64.3)	
N/A	60 (42.9)	80 (57.1)	
Peripheral vascular disease			
No	39 (48.8)	41 (51.3)	0.262
Yes	63 (38.2)	102 (61.8)	
N/D	50 (44.2)	63 (55.8)	
Duration of hospital stay	17.65 ± 17.12	22.98 ± 36.12	0.466
ICU admission			
No	97 (43.3)	127 (56.7)	0.915
Yes	53 (41.1)	76 (58.9)	
N/A	2 (40)	3 (60)	
Outcome			
Death	29 (38.7)	46 (61.3)	0.744
Discharged alive	113 (43.3)	148 (56.7)	
Transfer to another facility	2 (66.7)	1 (33.3)	
N/A	8 (42.1)	11 (57.9)	
Death among all patients			
Yes	29 (38.7)	46 (61.3)	0.455
No	123 (43.5)	160 (56.5)	
Death among amputated			0.532
No	104 (43.7)	134 (56.3)	
Yes	26 (39.4)	40 (60.6)	
Death cause			
Sepsis	12 (35.3)	22 (64.7)	0.374
Cardiac arrest	9 (60)	6 (40)	0.16
Brain compression	1 (100)	0 (0.0)	0.244
Septic shock	6 (37.5)	10 (62.5)	0.681
ESRD	1 (20)	4 (80)	0.306
More than one cause	7 (46.7)	8 (53.3)	0.736

## Discussion

COVID-19 has become a healthcare challenge across the world starting with the substantially decreasing chronic patient monitoring, management, and follow-up [5,6].

We aimed to determine the impact of COVID-19 on patients with DFUs over the same period in 2019-2020 (pre-lockdown) and 2020-2021 (post-lockdown) due to the COVID-19 pandemic.

Unlike the expected results this study found no significant difference in amputation rate and mortality before and during the pandemic compared with the existing studies [7-10], despite the avoidance of emergency department visits, relative procedure restrictions, postponing the routine visit, improper diet, nonadherence to medications, and physical inactivity during the lockdown [7,11].

The observations agreed with the results of a population-based cohort study in Ontario, Canada reported that the COVID-19 pandemic was not associated with increased limb loss in diabetic patients [8]. On the other hand, this finding is contrary to previous studies at a multidisciplinary center in China that reported that 11.4% of the hospitalized patients with a diabetic foot ulcer (DFU) undergo major amputation during the pandemic compared with 4.6% of pre-pandemic patients [9]. At a tertiary care center in Naples, Italy, a higher risk of amputation was observed by a factor of 2.5 during the COVID-19 lockdown among hospitalized patients with DFU [10].

Similarly, in a hospital in Chennai, India, the number of major amputations increased by 54% in the pandemic period compared with the pre-pandemic period [7]. There are possible explanations for more positive results observed in our tertiary center, which are as follows: The multidisciplinary approach and diabetic foot team plans, separation of our team into two groups with different working days to decrease the risk of whole team infections and maintain patient management during the crisis, and the importance of keeping in contact with our patients by shifting to the virtual clinic; another explanation may be that limited daily activities during lockdown contributed to lower diabetic foot ulcers, initiated by repetitive microtrauma to the foot and toes in the presence of neuropathy. This hypothesis corresponds with the value of proper footwear and offloading of pressure points that is supported by a recent international study [12].

Furthermore, we explored the clinical characteristics and the outcomes in-patients with diabetic foot ulceration (DFUs). Across time intervals during the pandemic, there were no consistent differences in demographic or comorbidities between individuals before and during the crisis, as these data showed that the most common comorbidities during the two periods were as follows: hypertension, chronic kidney disease and chronic vascular disease. Similarly, data from a large-scale, multi-center, retrospective study in Canada showed that approximately 95% of patients had hypertension, 38% ischemic heart disease and 15% end-stage renal disease [13].

Of note, we found a non-significant higher proportion of patients' emergency admission (43.9%, n=130) and a lower number of patients coming from regular outpatient access (35.7%, n=20) among the individuals admitted during the lockdown than those admitted during pre-lockdown. It is noteworthy that patients with diabetic foot had significant complications with acute lower limb ischemia (P-value=0.029) during the pandemic, as the COVID-19 pandemic suddenly interrupted patient foot education, diagnosis and treatment of foot complications due to the suspension of outpatient clinics and hospital visits [13,14].

Limitations

This study has a main limitation that could affect the results. There was a significant decrease in non-Saudi patients during the pandemic compared to the pre-pandemic period. One of the reasons for this is many of our patients in the hospital were illegal residents without medical insurance, and with the restrictions during the lockdown, only patients with official permission could go to the hospital. These difficulties led to delays in receiving appropriate management (Early vaccination, Tawakalna app).

## Conclusions

In conclusion, this study demonstrates that the COVID-19 pandemic was not associated with an increase in amputation rate along with mortality rate. As we emerged from a third severe wave of COVID-19, we noticed that the healthcare services that we provide during the pandemic, considering our hospital protocol and resources, have better outcomes in relative to other hospitals worldwide (resources). This showed a great impact on protection methods which eventually affect the results of our clinical research.

Anyhow the data emphasizes that healthcare access to diagnosed diabetic patients should continue at the standard level of care during pandemics, and that with the help of a multidisciplinary team to formulate compliance monitoring strategies. Moreover, we need continuing awareness about medication use, foot care, complications and to treat comorbidities along with COVID-19 infection, especially among the elderly who are already suffering from serious and critical infections in order to decrease the risk of amputation and mortality. Furthermore, we suggest undertaking studies on a larger scale by covering multiple institutions to determine a more accurate amputation rate and in-hospital death rate in the country.
